# Experimental Matching of Instances to Heuristics for Constraint Satisfaction Problems

**DOI:** 10.1155/2016/7349070

**Published:** 2016-02-01

**Authors:** Jorge Humberto Moreno-Scott, José Carlos Ortiz-Bayliss, Hugo Terashima-Marín, Santiago Enrique Conant-Pablos

**Affiliations:** National School of Engineering and Sciences, Tecnológico de Monterrey, Avenida Eugenio Garza Sada 2501 Sur, Colonia Tecnológico, 64849 Monterrey, NL, Mexico

## Abstract

Constraint satisfaction problems are of special interest for the artificial intelligence and operations research community due to their many applications. Although heuristics involved in solving these problems have largely been studied in the past, little is known about the relation between instances and the respective performance of the heuristics used to solve them. This paper focuses on both the exploration of the instance space to identify relations between instances and good performing heuristics and how to use such relations to improve the search. Firstly, the document describes a methodology to explore the instance space of constraint satisfaction problems and evaluate the corresponding performance of six variable ordering heuristics for such instances in order to find regions on the instance space where some heuristics outperform the others. Analyzing such regions favors the understanding of how these heuristics work and contribute to their improvement. Secondly, we use the information gathered from the first stage to predict the most suitable heuristic to use according to the features of the instance currently being solved. This approach proved to be competitive when compared against the heuristics applied in isolation on both randomly generated and structured instances of constraint satisfaction problems.

## 1. Introduction

Combinatorial problems are recurrent in artificial intelligence and related areas. The current literature contains a significant amount of work that has focused on designing and implementing methods that successfully solve these problems by combining the strengths of existing algorithms to improve the performance. Examples of these methods include dynamic algorithm portfolios [1–3], selection hyperheuristics [4–6], and instance specific algorithm configuration (ISAC) [7]. In general, all these methods manage a set of algorithms (solvers, heuristics, or strategies) and apply one that is suitable to the current problem state of the instance being solved. Although different names have been used in the literature, from this point on, we will refer to these methods as algorithm selectors.

Algorithm selectors relate instances to one suitable strategy to be used during the search, based on its historical performance on similar instances. These methods have proven to be reliable for solving a much wider set of instances than the algorithms they select from. These strategies keep a record of the historical performance of different algorithms on a set of solved instances in order to estimate, based on the similarity of the instances, the expected performance of such algorithms on unseen instances. To estimate how similar two instances are, the algorithm selectors compare the values of a set of features that characterize the instances. With this, algorithm selection strategies define and maintain a relation of instances to algorithms that is used to determine a suitable algorithm to be used when a new instance is presented to the system. Unfortunately, the internal representations of the relation between instances and algorithms are usually hardly interpretable by humans, making it difficult to understand how the algorithm selectors make their decisions.

In this investigation, we focus on analyzing the relation between instances and heuristics for constraint satisfaction problem (CSP), which is one of the most studied combinatorial problems in the literature. A CSP consists of a set of variables *v* ∈ *V* that contains the variables that need to be assigned a value from a corresponding domain *d*_*v*_, and there exists a set of constraints *C* that restricts the values a subset of variables can simultaneously take. The importance of CSPs lies in the fact that many combinatorial problems such as scheduling [8], radio link frequency assignment [9], and microcontroller selection/pin assignment [10] can be formulated as CSPs.

CSPs are usually solved by using backtracking-based algorithms [11]. Backtracking-based algorithms explore the space of solutions by using depth-first search, where every node in the search tree represents an assignment. The process starts with an empty variable assignment that is iteratively extended until obtaining a complete assignment that satisfies all the constraints or the instance is proven to be unsatisfiable [12]. These algorithms rely on a constructive approach that takes one variable at the time and consider only one value for it. Heuristics are usually applied to decide the next variable to instantiate and which value to use. These heuristics are commonly referred to as variable and value ordering heuristics, respectively. Once a variable is assigned a value, the search evaluates whether the assignment breaks one or more constraints. If that is the case, another value must be tried for such variable. If during the search any variable runs out of values, the algorithm backtracks and assigns a new value to the variable located at the backtracking position. Thus, the algorithm tries to undo the path once a failure has been detected by going back to upper levels until it gets to a variable where it can change its value and continue the search from that point. In general, the better the decisions of the heuristics, the smaller the cost of the search. But heuristics are problem dependent and then their performance may significantly vary from one instance to another.

This study analyzes the behaviour of six variable ordering heuristics to identify the most suitable ones for specific regions of the CSP instance space and also which ones should not be used on certain areas of the space. The hypothesis is that we can use information from the individual performance of various variable ordering heuristics on a set of instances to produce easy-to-interpret rules to predict the performance of such heuristics on unseen instances. The overall goal of this investigation is to use the information collected about heuristics and their performance on various instances to produce an algorithm selector that recommends the most suitable heuristic to use according to the features of the instance at hand in order to minimize the cost of the search.

This paper provides insights into how to answer two important questions for the community: (1) given a heuristic, on which instances is it likely to perform well? (2) Given an instance, which heuristics are likely to perform well? These questions are carefully addressed by an extensive experimental setup that includes the analysis of six variable ordering heuristics on more than two hundred thousand instances. Finally, derived from this analysis, we proposed a simple but useful algorithm selector that exploits the strengths of six different heuristics to improve the search. In summary, the main contributions of this paper are as follows:

- The analysis of the performance of six variable ordering heuristics on a large set of CSP instances by pointing out their strengths and limitations.
- A methodology to identify suitable and unsuitable regions of the CSP instance space for specific heuristics.
- Two algorithm selection strategies with an internal representation of the relation between instances and heuristics which are simple to interpret by humans. These algorithm selectors choose one suitable heuristic according to the features of the instance being solved and the information obtained from the historical performance of the heuristics on similar instances.
- The empirical evidence that including more than one heuristic when solving problem instances is not always beneficial for a heuristic selection strategy, as some heuristics may cancel the progress of others if used to solve the same problem at different stages of the search.

This paper is organized as follows.  Section 2 introduces relevant works on the analysis of algorithms and algorithm selection through an exploration of the instance space of various combinatorial problems. A detailed description of the heuristics considered for this investigation is provided in Section 3. The analysis of the performance of variable ordering heuristics on the CSP instance space is outlined in Section 4.  Section 5 describes the algorithm selection strategies proposed, the results obtained, and the discussion of these results. Finally, we present the conclusion and discuss some ideas for future work in Section 6.

## 2. Background and Related Work

In general, the task of selecting the most suitable algorithm for a particular problem is referred to as the algorithm selection problem [13] and this concept has been applied to various problems in the past few years. Stützle and Fernandes [14] collected a large amount of quadratic assignment problem (QAP) instances to conduct a systematic study of the performance of some algorithms according to the features of the instances. Smith-Miles [15] proposed a framework for analyzing the performance of various algorithms for QAP instances to get insights into the relationship between instance space features and the performance of the algorithms evaluated. In a subsequent study, Smith-Miles et al. analyzed the performance of heuristics for the scheduling problem by using a decision tree [16]. To conduct the analysis, 75000 scheduling instances were generated and solved by using two common scheduling heuristics. The authors used a self-organizing map to visualize the feature space and the corresponding performance of the heuristics, in order to get insights into the heuristic performance. More recently, Smith-Miles et al. compared the strengths and weaknesses of different optimization algorithms on a broad range of graph coloring instances [17].

Bischl et al. tackled the algorithm selection problem as a classification task based on an exploratory landscape analysis [18]. The authors used systematic sampling of the instances to collect a set of features and used those features to predict a well-performing algorithm (in terms of expected runtime) out of a given portfolio. One-sided support vector regression was used to solve the resulting learning problem. López-Camacho et al. [19] applied principal component analysis as a knowledge discovery method to gain understanding on the structure of bin packing problems and how it relates to the performance of various heuristics for this problem.

With regard to CSPs, Tsang and Kwan [20] introduced the idea of systematically relating instances to suitable algorithms, based on the features of those instances. In that study, the authors presented a survey of algorithms for solving CSPs and established the first ideas that suggested that it was possible to relate the formulation of a CSP to one adequate solving algorithm for that formulation. This idea supports more recent algorithm selection approaches, like the ones described in the following lines. Ortiz-Bayliss et al. [21] studied the performance of two variable ordering heuristics on a large set of CSP instances. In their analysis, the authors found preliminary evidence that supports the idea that some heuristics for CSP can indeed be used in collaborative fashion to improve the search.

A preliminary idea of this investigation was presented by Moreno-Scott et al. [22], where three heuristics were analyzed at a much smaller detail than the one presented in this document. This investigation extends the previous study by including three more variable ordering heuristics into the analysis, formalizing the instance space characterization to help us identify regions of difficult and easy instances, and describing a simple but useful way to use the information from the analysis to predict a suitable heuristic to improve the search.

There is also a growing interest in the generation of particularly difficult or easy instances for testing algorithms, in order to understand when they are preferable to others. Usually, the generation of such instances is done by using evolutionary computation. The idea is to construct generation models that provide a more direct method for studying the relative strengths and weaknesses of each algorithm. Smith-Miles et al. proposed the use of an evolutionary algorithm to produce distinct classes of traveling salesman problem (TSP) instances that are intentionally easy or hard for certain algorithms [23, 24]. In their analysis, a comprehensive set of features is used to characterize the instances. By using the information gathered from the performance of these algorithms on the set of instances, the authors proposed a prediction algorithm that presents high accuracy on unseen instances for predicting search effort as well as identifying the algorithm likely to perform best.

For CSPs, van Hemert [25, 26] proposed a genetic algorithm to produce instances that are difficult to solve. van Hemert's model maintains a population of binary CSPs of which it changes the structure over time. Its genetic operators modify the conflicts between the pairs of variables. Under this approach, the set of variables and their domains are kept unchanged during the whole process. Thus, only the ratio of forbidden pairs of values can vary as a result of the evolutionary process. As part of the generation process, the algorithm requires solving the instances to evaluate their fitness. Moreno-Scott et al. [22] used van Hemert's model to generate extremely hard instances for specific variable ordering heuristics. Among their main findings, the authors confirmed that instances that are hard to solve for some heuristics may not be hard for others.

## 3. Variable Ordering Heuristics

Six dynamic variable ordering heuristics were considered for this investigation due to their performance in previous studies [5, 27–29]. Each heuristic assigns a score to the variables in the instance being solved, based on a specific criterion as the search progresses. According to its particular strategy, every time a variable is to be selected for instantiation the heuristic sorts the variables by their score in ascending or descending order, and the first variable in the sorted list is selected for instantiation. In all cases, ties among variables are broken by using the lexical order of the names of the variables. Regarding the order in which the values of the selected variables are tried, values are always tried in the default order in which they appear in the domain of the variable to instantiate.

Because instantiating a variable changes the problem state (as domains and constraints are updated), the scores given by any of the heuristics to the remaining variables are likely to change at different stages of the search. For this reason, the ordering of the variables is dynamic, deciding which variable to instantiate considering the current problem state and the current scores given by a particular heuristic.

The following lines describe the variable ordering heuristics used in this work:

(i) *Minimum Domain (DOM)*. DOM [30] instantiates first the variable with the fewest values in its domain. Then, DOM selects the variable that minimizes *d*_*v*_ among all the variables, where *d*_*v*_ is the current domain size of variable *v*.
(ii) *Maximum Degree (DEG)*. This heuristic considers the degree deg_*v*_ of the variables to decide which one to instantiate before the others. The degree of a variable is calculated as the current number of constraints where the variable is involved. Thus, DEG instantiates first the variable with the largest deg_*v*_ [31].
(iii) *Minimum Domain over Maximum Degree (DOMDEG)*. DOMDEG tries first the variable that minimizes the quotient *d*_*v*_/deg_*v*_ among the remaining variables in the instance [32].
(iv) *Minimum Solution Density (RHO)*. This heuristic is based on the approximated calculation of the solution density of the CSP instance, *ρ* [5, 28]. Let *C*_*v*_ indicate the constraints in which variable *v* is involved. Then, RHO will instantiate first the variable that minimizes *ρ*_*v*_ = ∏_*c*∈*C*_*v*__(1 − *p*_*c*_), where *p*_*c*_ is the current fraction of forbidden pairs of values among constraint *c*.
(v) *Minimum Expected Solutions (SOL)*. SOL instantiates the variables in such a way that the resulting subproblem contains the maximum number of expected solutions [5, 28]. To do so, the search branches on the variable that minimizes sol_*v*_ = *d*_*v*_ × *ρ*_*v*_.
(vi) *Maximum Conflicts (MXC)*. This heuristic prefers the variable that maximizes the number of conflicts where it is currently involved [33]. A conflict represents a pair of values that is not allowed for two variables at the same time. A constraint between two variables *x* and *y* may contain zero or more conflicts (up to *d*_*x*_ × *d*_*y*_). The larger the number of conflicts in a constraint is, the more difficult it is to satisfy. MXC will select first the variable that maximizes *cf*_*v*_ = ∑_*c*∈*C*_*v*__(*p*_*c*_).

**Figure 1:**
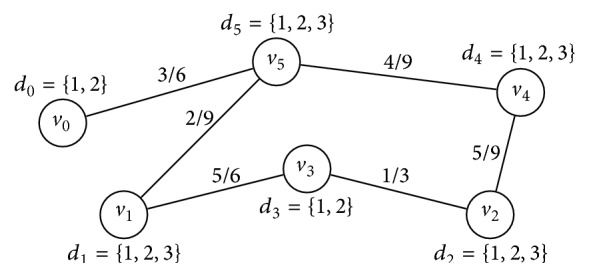
A CSP instance with six variables and domains of two and three values. Nodes represent variables and edges represent constraints. The values on edges indicate the fraction of forbidden pairs of values per constraint.

To help clarify how these heuristics make their decisions, a simple CSP instance is depicted in Figure 1 and analyzed by using each heuristic.  Table 1 presents the scores given to the variables according to each heuristic. As the reader may observe, there are cases where different heuristics select the same variable. In this example, DOMDEG, RHO, and SOL will instantiate *v*_3_ first, while DOM, DEG, and MXC will prefer *v*_0_, *v*_5_, and *v*_4_, respectively.

## 4. An Experimental Evaluation of Variable Ordering Heuristics

In this section, we explored the CSP instance space by generating and solving a vast set of randomly generated instances. The instances were solved by using a backtracking-based algorithm where the ordering of the variables was defined dynamically by using the heuristics described in Section 3. The CSP solver used in all the experiments in this investigation was fully implemented in Java. To speed up the search, the solver incorporates backjumping [34] and constraint propagation [35]. All the experiments were conducted on an AMD 4.0 GHz 8-Core Windows machine with 32 GB of memory.

In this work, we have focused exclusively on binary CSP instances with constraints defined in extension (the forbidden pairs of values in the constraints are explicitly listed). Although the ideal case would be to support instances with constraints of *n*-arity (for *n* > 2), at this point, it is difficult to define features to characterize such instances. Every instance in this investigation is characterized by using two well-studied binary CSP features: the constraint density (*p*_1_) and the constraint tightness (*p*_2_). The constraint density of a CSP instance is calculated as

(1) $$\begin{array}{c} p_{1}=\frac{2\left|C\right|}{\left|V\right|\left(\left|V\right|-1\right)}, \end{array}$$

where |*C*| and |*V*| represent the number of constraints and variables in the instance, respectively. The constraint tightness of an instance is calculated as

(2) $$\begin{array}{c} p_{2}=\frac{1}{\left|C\right|}\underset{c\in C}{\sum }p_{c}, \end{array}$$

where *p*_*c*_ is the fraction of forbidden pairs of values in constraint *c*. For example, the constraint density and tightness of the example CSP shown in Figure 1 are 0.4 and 0.4815, respectively. Although other features to describe binary CSPs are available in the literature, we have decided to work with *p*_1_ and *p*_2_ because they have been widely used in the past and proven to provide an easy-to-interpret description of the instances.

Two main sources of CSP instances exist: randomly generated instances and structured ones from real-world applications. Structured instances from real-world applications are usually the best source but unfortunately are usually short in supply. Thus, instance generators provide a good additional source for testing algorithms. These generators have the advantage of providing a precise control over the problem features, such as size and expected hardness [36, 37], and facilitate the systematic analysis of algorithms [38].

In this investigation, we systematically explored the CSP instance space by using the instance generation model B [39] and analyzed the performance of the six heuristics on the instances produced. Model B divides the generation process into two stages (see Algorithm 1): the generation of the constraints between the variables and the generation of the forbidden pairs of values among the variables linked by a constraint. Although other generation models exist [25, 40–42], we have opted for model B for its simplicity and accuracy in producing instances with the precise values of density and tightness we required for detailed sampling of the CSP instance space.

We produced a grid of 50 × 50 sampling points distributed on the instance space *p*_1_ × *p*_2_. The points in this grid are separated by steps of 0.02 in each axis, resulting in a grid of 2500 uniformly distributed sampling points. For each point in the grid, we generated 50 instances of 20 variables and 10 values in their domains, with the values of *p*_1_ and *p*_2_ of the corresponding sampling point.

Once the grid was produced, we used the variable ordering heuristics described in Section 3 to solve all the instances. For the purpose of this investigation, an instance is considered solved when the first solution is found or the instance is proven unsatisfiable. Because of this, any solution is equally desirable. To estimate the cost of the search, we used the number of consistency checks executed during the search. Every time a constraint was revised, the counter for consistency checks increased by one. Thus, the more the revisions of the constraints, the higher the cost of the search. In total, 125,000 instances were generated to construct the grid of instances and each one of these instances was solved six times, one time per heuristic analyzed. At this point, it is important to stress the difference between the average cost per sampling point and cost of solving a specific instance. The cost of solving an instance is the number of consistency checks required to solve an instance by using one particular heuristic. Consequently, the cost of a sampling point is the average cost of one particular heuristic on the 50 instances generated for such sampling point.

**Figure 2:**
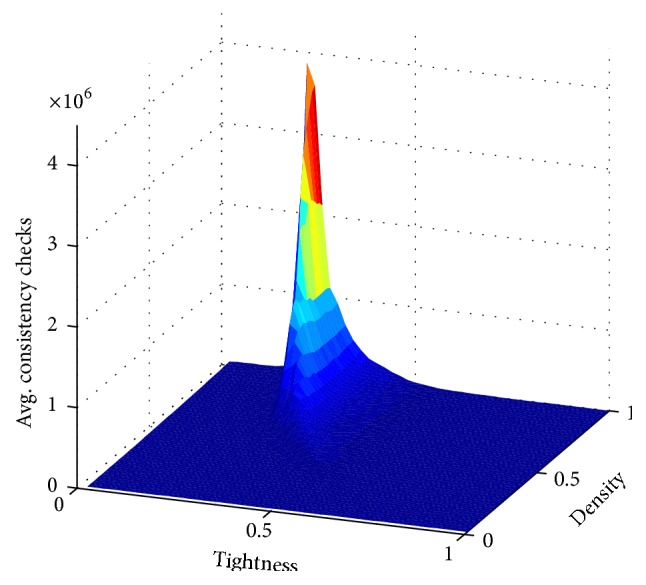
Average consistency checks per sampling point in the grid required by SOL.

**Figure 3:**
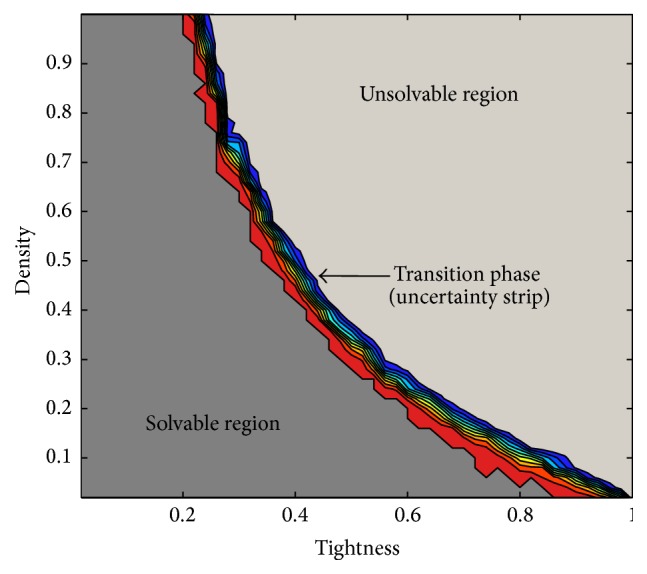
Phase transition for the grid of instances with 20 variables and 10 values per domain.

Figure 2 shows the landscape of the instance space according to the average consistency checks required by SOL to solve the 50 instances in every sampling point in the grid. The surfaces obtained for the rest of the heuristics are very similar in shape to Figure 2, but with differences in the number of consistency checks required to solve the instances. Independently of the heuristic used to solve the instances, a phase transition phenomenon is observed [43–45]. This phase transition region is where instances abruptly stop being satisfiable and contains, in average, the most difficult to solve instances, regardless of the heuristic used. Figure 2 offers a view of the instance space that illustrates the phase transition region for the grid of instances produced. In practice, the phase transition region is depicted as a narrow strip in which the maximum uncertainty about the satisfiability of the instances is reached (Figure 3).

Table 2 identifies the values of *p*_1_ and *p*_2_ of the sampling points for which each of the heuristics required the maximum average consistency checks. Unsurprisingly, the highest average costs of the six heuristics are located at the same point (*p*_1_ = 1.0 and *p*_2_ = 0.22). These values of *p*_1_ and *p*_2_ correspond to the peak in average consistency checks on the phase transition region shown in Figure 3. This observation is not new and only confirms what we already know about the phase transition: in average, it contains the hardest-to-solve instances for any backtracking-based method. But there is more to learn from these results. For example, there is a large difference between the consistency checks required by each heuristic for this particularly hard sampling point. Despite this point being difficult for all the heuristics, one of them is better than the others, showing that there is something that can be learnt to improve the search (even for points in the most difficult to solve region).

In this sampling point (and in general for all the points in the phase transition region), SOL proved to be a very competent heuristic. The average cost per point on the phase transition region is usually smaller for SOL than for the other heuristics. This means that for the 50 instances in each point on the hardest-to-solve region SOL required, in average, the fewest consistency checks to solve those specific instances. The fact that SOL was the best average heuristic for the specific region of the instance space where the most difficult instances take place must not be interpreted as a proof that SOL is the best absolute heuristic over the whole space. As we will show in Section 4.1, heuristics are specialists for specific regions in the instance space and outside those regions their performance decreases significantly.

Table 2 also includes the maximum and minimum consistency checks required by each heuristic in the point (*p*_1_ = 1.0, *p*_2_ = 0.22). It is interesting to observe that the difference between the hardest and easiest instance per heuristic in such point is huge. The former indicates that it is possible to find a mixture of hard and easy instances, even for points located inside the phase transition region.

### 4.1. Matching Instances to Variable Ordering Heuristics

**Figure 4:**
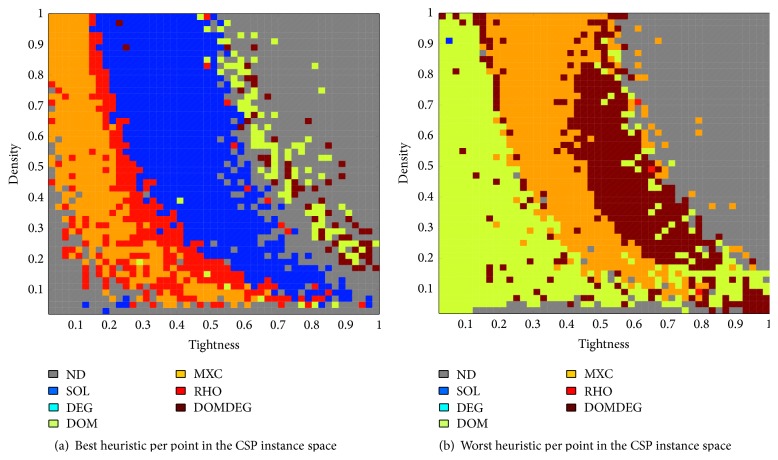
Best and worst heuristics in the CSP instance space.

We collected all the information about the average performance of each of the six heuristics on every point in the grid. Then, we compared their results to produce mapping between regions of the instance space and the expected performance of the heuristics. This information is summarized graphically in Figure 4(a), where the best performer per point in the grid among the six heuristics is shown. The smaller the average cost per point, the better the performance of the heuristic. Although there is no dominant heuristic for all the instances in the grid, some regions seem more suitable for certain heuristics than for others (a heuristic *A* is said to dominate heuristic *B* on point *P* if the average cost of *A* is lower than the average cost of *B* for point *P*). The regions of dominance seem to somehow follow the shape of the phase transition curve. It is interesting to observe that SOL clearly dominates the other heuristics on the phase transition region. But this situation dramatically changes as we move away from this region. As we approach the region where all the instances are satisfiable (on the left side of the phase transition region), some strips of dominance become visible: regions where RHO and MXC obtain the best performance (from right to left). On the right side of the phase transition, where most of the instances are unsatisfiable, there is small dominance of DOM and DOMDEG, which indicates that these heuristics are useful when the instances are unsatisfiable. However, in most of the unsatisfiable region of the instance space, two or more heuristics obtain the best performance (there is no single dominant heuristic). This is illustrated in Figure 4(a) with the label ND for “no dominance.” Similarly, on the bottom left corner of the graph, where the problems are easy and satisfiable, there is no dominance of one heuristic over the others because most of the instances are trivially solved. Note that heuristic DEG does not appear in the figure, since it is never the dominant heuristic for any of the points in the grid.

We have identified the most suitable heuristics for specific regions of the instance space, but it is also relevant to identify the regions where it may not be wise to apply a particular heuristic. For this reason, Figure 4(b) shows a similar analysis to the previous one but focused on the worst heuristic per point. As in the previous case, some patterns of dominance are clearly visible. At the region where the problems are satisfiable, DOM is the worst heuristic, as it is dominated by the other five heuristics. This result makes sense, as DOM proved to be a specialist only for some unsatisfiable instances (see Figure 4(a)). MXC is the worst choice for points close to the phase transition region. There is a narrow strip that follows the phase transition curve, on the unsatisfiable region where DOMDEG presents its worst performance. Finally, on the right side of the space where the instances are unsatisfiable, no heuristic is always dominated by the others. This means that at least two heuristics showed the same poor performance.

We have obtained information about the average performance of the heuristics on the instance space. This allowed us to identify regions where certain heuristics should be used and which ones should not. But what is the performance of the heuristics on the 50 instances of each particular point in the grid? For example, Table 3 presents the results obtained by each of the six heuristics on the 50 instances contained in the point (*p*_1_ = 1.0, *p*_2_ = 0.22), where the highest average cost in the grid was achieved. The best average heuristic for this point, SOL, obtained the lowest cost in 42 instances. MXC, as expected, obtained the worst costs in 46 instances. If we consider only the average performance of these heuristics, it might seem obvious that MXC should never be used for instances with these values of *p*_1_ and *p*_2_. However, MXC obtained the best result in one isolated case (instance 27) with an impressive performance three times better than SOL. This indicates that even though we can estimate the average performance of the heuristics on the instances based on their values of *p*_1_ and *p*_2_, there is no guarantee that we will always select the best option for all instances as there are cases where an apparently suboptimal performer may be a really useful solving option. An important consequence of this result is that although we can use *p*_1_ and *p*_2_ to properly estimate the average expected performance of the heuristics, more features are needed to fully distinguish extremely strange cases where one usually bad heuristic should be preferred. Including additional features represents an important step towards improving the mapping between instances and heuristics in the future.

## 5. Using the Matching of Instances to Heuristics to Improve the Search: A Heuristic Selection Approach

In previous sections, we described the mapping of instances to heuristics obtained by the systematic exploration of the instance space. We have found that some heuristics are, in average, better than others for certain regions of the instance space. In this part of the investigation, we were interested in using the information gathered from the previous experiments to see whether it is possible to use such information to improve the search when tested on a wider set of instances, on both randomly generated and structured ones. The assumption is that, by selecting the right heuristic according to the initial problem state, we can reduce the cost of the search and show a better performance than with the variable ordering heuristics applied in the traditional fashion.

We proposed two heuristic selection strategies for this experiment. These strategies consider the current conditions of the problem, described exclusively by the values of *p*_1_ and *p*_2_ of the instance to solve, and apply the best heuristic for those conditions based on the patterns depicted in Figure 4(a). The heuristic selection strategies consist of a grid of easy-to-interpret rules, one rule per cell of the mapped instance space in the form (*p*_1_, *p*_2_) → heuristic. The rule with the smallest Euclidean distance from its condition to the current values of *p*_1_ and *p*_2_ of the instance to be solved is the one that fires and determines the heuristic to be applied. In other words, the values of *p*_1_ and *p*_2_ of the instance to solve are used to place the instance on the grid shown in Figure 4(a) and the best average heuristic for that point is used. The first heuristic selector (SHS) is static and it only evaluates the state of the instances at the beginning of the search. This results in a selection strategy that only makes one decision per instance and once the decision is made, the same heuristic is used to solve the whole instance, from the beginning to the end. The second heuristic selector (DHS) is dynamic. DHS evaluates the problem state every time a variable is to be assigned and then different heuristics are applied as part of the solving process. Although both heuristic selectors use the same information to make their decisions, they use such information differently. Two consequences of the difference in the use of the information are observable. First, the time for making the decisions is slightly shorter for SHS, as it only evaluates the problem state once. In this work, the additional time required by DHS to compute the problem state was not an issue, as the set of features to characterize the instances is small and the features are easy to compute. But in other cases, it may represent significant delays if more hard-to-compute features are considered. The second aspect to consider is the interaction of heuristics during the search. While some heuristics may contribute to others and improve the search, there is also a risk that some heuristics cancel the effect of others, taking the search into unpromising areas of exploration. Thus, we are likely to observe higher variance in the results if DHS is used.

We do not claim that the heuristic selection strategies described in this document are the best models among the heuristic selection methods described in the literature. In fact, a comparison between models would be a good idea for future work. At this point, our only idea is to show that there is actually a practical use of the information obtained from the analysis of the relation between instances and the performance of heuristics to solve unseen CSP instances.

### 5.1. Testing the Heuristic Selectors on Randomly Generated Instances

The heuristic selectors described before were tested on three additional grids of instances generated exclusively for testing purposes. Each one of these new grids contains 2500 sampling points (as the one used for exploring the performance of the heuristics) with 10 instances per point. The instances in these grids contain 20 variables and 10 values in their domains. In total, 75,000 additional instances were generated for the testing phase. The analysis of the heuristic selector is based on two criteria: the percentage of points where the heuristic selector is better than each heuristic among all the points per grid and the actual reduction in consistency checks obtained by using the heuristic selector with respect to the heuristics among all the instances in each test grid. The first criterion estimates how stable the heuristic selector is, while the second one provides an idea of the benefit of using this method over the single heuristics.

Tables 4 and 5 present the percentage of sampling points in the instance space in which the heuristic selectors SHS and DHS performed better than each particular heuristic. Except for DOM (which was the worst average performer for the test grids), SHS always obtained better results than DHS in the head-to-head comparison against the heuristics. Regardless of the differences between SHS and DHS, it is interesting to note the high percentage of instances where the heuristic selection strategies dominate SOL (which was the best performer for the instances within the phase transition region). Although SOL is a good heuristic for hard-to-solve instances, the percentage of instances where the selectors are better than SOL is larger than the percentage obtained for the other heuristics. The reason for this is that SOL is not a competent heuristic for solving not-so-hard instances (which cover around 60% of the instance space).

Tables 6 and 7 complement the information about the performance of SHS and DHS. In these tables, we indicate the percentage of consistency checks saved by using SHS and DHS with respect to the heuristics applied in isolation. The reason for this comparison is that it is not enough to know that the heuristic selectors reduced the number of consistency checks required by a specific variable ordering heuristic; we are also interested in knowing how many consistency checks we can save by using these strategies. From Tables 6 and 7, we can clearly observe what we discussed before about SOL and its performance inside the phase transition region. SOL is a very reliable heuristic for the region where the hardest instances occur and as a consequence, the differences between the best and worst performer are huge, stressing the consequences of bad choices. Outside that region, the number of consistency checks is significantly lower for all the heuristics, resulting in smaller differences between the best and the worst performer (which reduces the impact of bad choices). Although SOL is a specialist for the region where the phase transition occurs, SHS is able to reduce the number of consistency checks of this heuristic in more than 1.5% for all the test instances. The performance of SHS, when compared to the other heuristics, is outstanding, but it is mainly because of the good performance of this selector on the hardest-to-solve instances. On the other hand, the dynamic selection of heuristics by DHS was not as effective as the strategy of SHS. Although DHS is able to obtain better results than SOL in around 65% of the test instances, the cost of using DHS to solve all the instances increases the number of consistency checks with respect to SOL in around 9.16% consistency checks.

In average, SHS performed better than DHS among the test grids used in this investigation. The results confirm that, for the instances used in this investigation, changing to a different heuristic once the search has started is not as beneficial as staying with a suitable initial choice of heuristic (choice based on what we know about the instances and the heuristics). The reason for this is that the patterns shown in Figure 4(a) estimate the best performer per instance assuming that the same heuristic is used from start to end. Because the patterns do not capture any information about the changes of heuristics as the search progresses, the dynamic selector is working on a pattern that may not be valid for making its decisions in the best way.

### 5.2. Testing SHS on Structured Instances

We have confirmed the idea that, by using the methodology proposed, it is possible to accurately predict a suitable heuristic for solving instances similar to the ones used for finding the relation between instances and heuristics. The historical information about the heuristics was collected from randomly generated instances to produce one static heuristic selection strategy that, according to the initial features of the instance to solve, decided the most suitable heuristic to apply. Although the results seem encouraging at this point, it is difficult, based only on the results obtained for randomly generated instances, to visualize how well the relation between instances and heuristics obtained could scale to larger structured instances.

**Figure 5:**
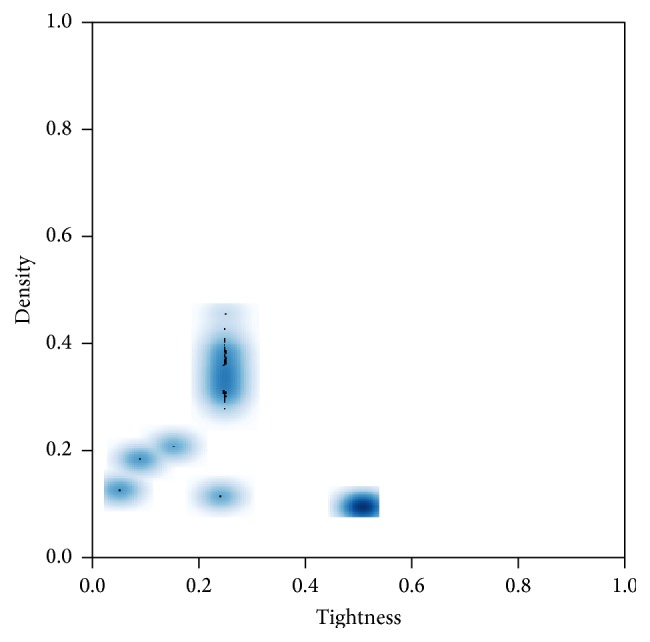
Density scatter plot for the structured instances used in this investigation (darker regions indicate a higher concentration of instances).

For this reason, we applied the best heuristic selector from the ones described before to solve a set of 250 instances with very different properties to the ones used for obtaining the patterns of use. Thus, the static heuristic selector, SHS, was used for the rest of the experiments. Contrary to the instances used so far in this investigation, the constraints in the instances used for this experiment contain some structure. For this investigation, we took and combined six files from a public repository (the public repository can be accessed at http://www.cril.univ-artois.fr/~lecoutre/benchmarks.html): composed-25-10-20.tgz, composed-75-1-80.tgz, ehi-85.tgz, geom.tgz, QCP-10.tgz, and QCP-15.tgz. Files QCP-10.tgz and QCP-15.tgz contain 15 instances each, with 100 and 225 variables, respectively, and nonuniform domains. The 20 instances from files composed-25-10-20.tgz and composed-75-1-80.tgz are composed of a main underconstrained fragment and some auxiliary fragments [46] and contain 105 and 83 variables, respectively, and 10 values in the domain of each variable. File ehi-85.tgz contains 100 3-SAT unsatisfiable instances represented as binary CSPs. The equivalent binary instances contain 297 variables and eight values in their domains. File geom.tgz contains 100 geometrical instances (instead of a density parameter, a distance parameter is used to define the distribution of the constraints among the instance) with 50 variables and 20 values in their domains.  Figure 5 presents the distribution of these structured instances on the instance space. In this case, we do not have the flexibility of the random generation model and as a result, most of the instances are distributed over some specific regions in the instance space.

When we compared the performance of SHS against each heuristic on this set of structured instances, we observed that the good performance shown on randomly generated instances is also presented in the set of structured ones. SHS dominates SOL in 51.83% of the instances and saves 19.22% consistency checks with respect to this heuristic. The results are better for the other heuristics. For example, in the case of DEG and MXC, the heuristic selection strategy dominates these heuristics in 76.73% and 91.84% of the instances, respectively, and reduces the number of consistency checks by 71.62% and 74.82%, with respect to each of these heuristics.

**Figure 6:**
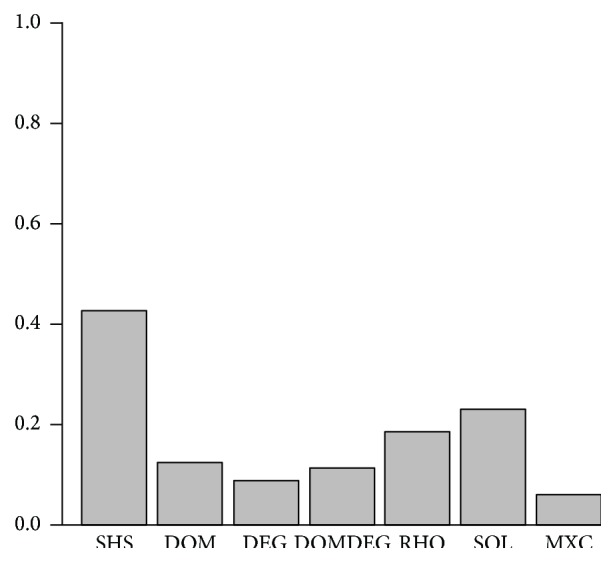
Percentage of instances on the set of structured instances where each method required the minimum number of consistency checks.

For this experiment, we were also interested in a more challenging comparison. For this reason, we tested SHS against the best possible result obtained from the six heuristics.  Figure 6 presents the percentage of instances where each method obtained the minimum number of consistency checks per instance. For some instances, two or more methods are tied with the minimum number of consistency checks. For this reason, when we sum up the percentages where each method required the minimum consistency checks, the result is greater than 100%.

The results depicted in Figure 6 remark the potential of the heuristic selection strategy proposed. By using SHS, we can, in more than 40% of the instances, obtain a cost comparable to the best possible one obtained with any of the six heuristics. This is more than we can achieve by using any of the heuristics applied in isolation (SOL is the closest heuristic in performance, achieving the minimum cost in 23% of the instances).

Predicting the best heuristic only in around 40% is sufficient to overcome the performance of the six heuristics evaluated in this investigation but gives plenty of room for improvement in the future. The small percentage of instances where the prediction is accurate is a consequence of the change in the type of instances used. As heuristics may present a different behaviour on randomly generated instances with respect to structured ones, the rules obtained for randomly generated instances may not be accurate for structured instances. We are aware of this situation and recognize that, for properly solving structured instances, the selectors should use information from similar instances. But with this experiment, we have proven that it is possible to improve the search by using the information about the historical performance of heuristics on instances that may not correspond to the same types used for extracting the information and producing the rules of application.

### 5.3. Comparison of Performance with Other Dynamic Heuristics

We have discussed the performance of SHS on different structured instances with respect to the heuristics available for the heuristic selector. In this experiment, we compare the performance of SHS versus the other two reliable dynamic ordering heuristics that are not available for the heuristic selector: activity-based search (ABS) [47] and weighted degree (WDEG) [48].

**Figure 7:**
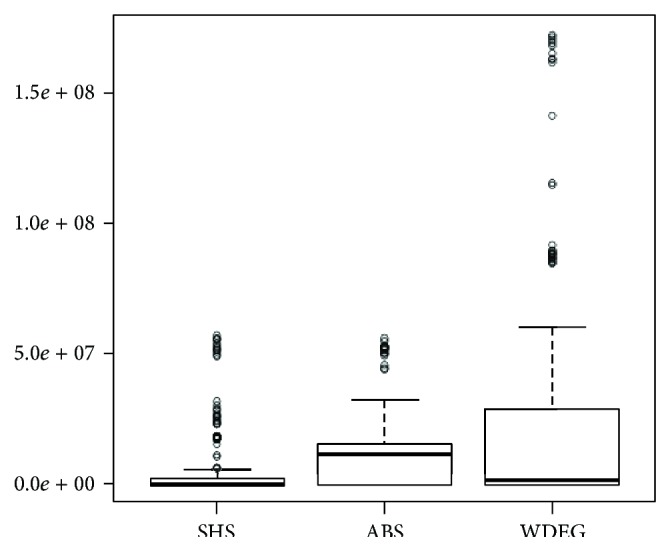
Performance of SHS, ABS, and WDEG on the set of structured instances.

The performance of the three methods is depicted in Figure 7. From this figure, we can observe that the median of SHS in the set of structured instances is lower than the median of ABS, but very similar to the one of WDEG. But the variance is higher for WDEG than for SHS and ABS.

Although the statistical evidence is insufficient to claim that SHS is better than ABS and WDEG (by using a unilateral paired *t*-test with 5% of significance), there are important savings in the cost of the search by using SHS that are worth discussing. By using SHS, we require, in average, 44.18% less consistency checks than ABS and 66.86% less consistency checks than WDEG on the set of structured instances. We are aware that the performance of the methods discussed may change if other sets of instances are used, but at this point, the results confirm that SHS is capable of competing against other reliable heuristics that were not part of the selection process.

## 6. Conclusion

This paper described a methodology to characterize the CSP instance space in order to analyze the performance of different variable ordering heuristics. This analysis allowed us to locate regions where some heuristics are better than others and also regions where some of these heuristics should not be used. The results confirmed that a large fraction of hard-to-solve instances are located on the same region of the instance space regardless of the heuristic used. But we also found evidence that, even for those regions, some heuristics are more desirable than others and that we can use such evidence to improve the search.

We identified regions in the instance space (characterized by the constraint density and tightness) where one heuristic dominates the others in average performance, but there is no absolute dominance among all the instances generated for specific values of density and tightness. For example, the best average heuristic for a set of instances can sometimes be defeated by an apparently weaker heuristic for some exact region of the instance space. We think that more features are required to identify such unusual situations and properly characterize those cases.

The instance space characterization provided an opportunity for understanding how heuristics work since it allowed us to identify where a heuristic should be used or avoided. Among the heuristics studied, SOL proved to be the most competent one for the region where the hardest average instances occur. An explanation for this good performance relies on the way different aspects of the problem are analyzed by the heuristic. While heuristics such as DOM, DEG, and MXC focus only on one aspect of the problem to decide the next variable to instantiate (the domain size, the degree, and the number of conflicts, resp.), RHO considers a mixture of two of them (the degree and the proportion of conflicts). For this reason, RHO is, in general, better than DOM, DEG, and MXC. But including more information may not always result in a better average performance. For example, DOM/DEG also combines information about the domain size and the degree of the variables but it does not seem to perform as well as RHO. Then, the way the different aspects of the problem are combined is also related to the performance of the heuristics. When we look at SOL, we observe that it is basically a revision of RHO, which is by itself a competent heuristic that considers two aspects of the problem. SOL extends RHO and improves its performance by including information about the domain size.

Although this information is by itself relevant and useful, we wanted to show that it can indeed be used to improve the search. By using the information obtained from the analysis of the heuristics and the instance space, we produced matching from instances to heuristics that was transparently translated into a heuristic selection strategy. This strategy was implemented by using the patterns obtained from the exploration of the performance of the heuristics on the CSP instance space. It is important to stress that the information the selector uses to make its decisions is easily interpretable by humans. This has various advantages, being among the most important ones that it is possible to visualize the relative strengths and weaknesses of these heuristics, allowing a more reliable heuristic performance prediction.

Among the two heuristic selectors proposed, the static one proved to be reliable and competitive for solving instances, both randomly generated and structured ones, as it improved the results obtained by any of the variable ordering heuristics when applied in isolation. The results confirmed our initial idea that the information obtained from the historical performance of heuristics on a set of instances can be used to improve the search (even when using such a simple strategy like the one described in this document). Now that we have confirmed our initial ideas, we can consider extending the matching process by including more features and heuristics and designing a more robust heuristic selection strategy.

As future work, we are interested in extending our investigation to explore other problem domains. Although the methodology described in this paper is focused on exploring the CSP instance space to predict the performance of some heuristics on such instances, it is not limited to this particular problem domain. The same model can be used on other domains provided that a systematic way to produce instances (to generate instances in a vast region of the instance space) and a suitable representation exist. Finally, we would like to use the information gathered from the mapping of heuristics to instances to produce more robust heuristic selectors that make better use of the information on the performance of the heuristics.

## Figures and Tables

**Algorithm 1:**
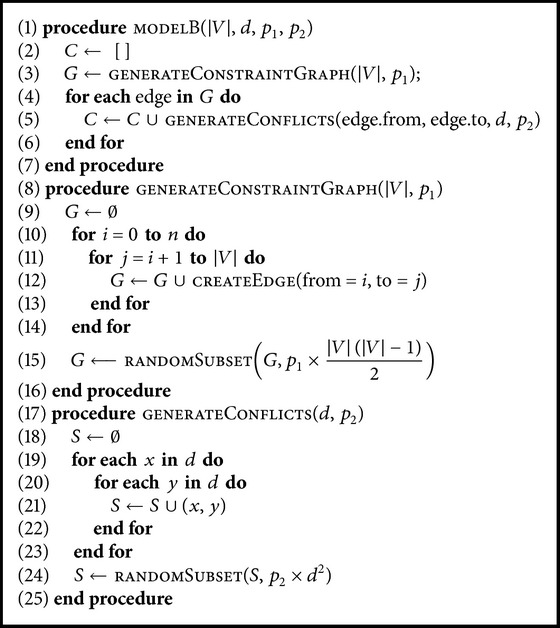
Instance generation model B.

**Table 1:** Scores given to the variables in the CSP instance depicted in Figure 1 by each one of the six heuristics. Values in bold indicate the scores preferred by each heuristic and ties are broken by using the lexical ordering on the names of the variables.

	DOM *d*_*v*_	DEG deg_*v*_	DOMDEG *d*_*v*_/deg_*v*_	RHO ρ_*v*_	SOL sol_*v*_	MXC *cf*_*v*_
*v*_0_	** 2**	1	2	0.5	1	3
*v*_1_	3	2	1.5	0.1296	0.3888	7
*v*_2_	3	2	1.5	0.2962	0.8886	6
*v*_3_	** 2**	2	** 1**	** 0.1111**	** 0.2222**	6
*v*_4_	3	2	1.5	0.2469	0.7407	** 9**
*v*_5_	3	** 3**	** 1**	0.2160	0.6480	** 9**

Selected	*v*_0_	*v*_5_	*v*_3_	*v*_3_	*v*_3_	*v*_4_

**Table 2:** Values of *p*_1_ and *p*_2_ of the points in the grid that, in average, required the largest number of consistency checks for each specific heuristic.

Heuristic	*p*_1_	*p*_2_	Consistency checks (millions)		
			(Avg.)	(Max.)	(Min.)
SOL	1.00	0.22	3.87	8.44	0.13
DEG	1.00	0.22	11.17	25.89	0.26
DOM	1.00	0.22	4.76	10.33	0.16
MXC	1.00	0.22	16.14	37.27	0.04
RHO	1.00	0.22	4.86	9.99	0.20
DOMDEG	1.00	0.22	11.17	25.89	0.26

**Table 3:** Consistency checks required by each heuristic at point (*p*_1_ = 1.0, *p*_2_ = 0.22). The best and worst result per instance are indicated with symbols ↓ and ↑, respectively.

Instance ID	Consistency checks (millions)					
	SOL	DEG	DOM	MXC	RHO	DOMDEG
0	↓ 2.41	6.02	2.61	↑ 6.10	3.40	6.02
1	↓ 6.91	17.65	8.23	↑ 27.99	9.43	17.65
2	↓ 7.06	23.22	8.07	↑ 26.42	8.54	23.22
3	↓ 6.98	19.95	9.21	↑ 30.94	9.50	19.95
4	↓ 4.64	15.14	6.02	↑ 16.80	7.14	15.14
5	↓ 6.91	18.43	8.56	↑ 30.52	9.03	18.43
6	↓ 1.14	↑ 5.09	2.33	4.17	1.22	↑ 5.09
7	↓ 7.56	24.24	9.43	↑ 37.27	9.42	24.24
8	1.64	↑ 3.96	↓ 1.45	3.76	1.93	↑ 3.96
9	↓ 2.05	4.62	2.24	↑ 5.49	2.63	4.62
10	↓ 6.64	19.08	8.16	↑ 29.92	8.27	19.08
11	↓ 6.19	15.26	7.70	↑ 25.76	7.68	15.26
12	↓ 6.06	19.97	10.33	↑ 31.11	9.13	19.97
13	↓ 7.11	25.89	8.62	↑ 32.52	8.77	25.89
14	↓ 1.44	3.91	1.93	↑ 7.25	1.57	3.91
15	1.68	3.30	1.63	↑ 4.70	↓ 1.41	3.30
16	5.89	13.46	6.83	↑ 22.24	↓ 5.78	13.46
17	↓ 1.40	3.64	1.71	↑ 5.02	1.64	3.64
18	↓ 1.88	8.57	2.81	↑ 8.64	2.41	8.57
19	↓ 4.98	↑ 17.18	7.27	16.75	5.89	↑ 17.18
20	↓ 4.46	10.48	5.58	↑ 16.08	5.17	10.48
21	↓ 1.07	2.52	1.35	↑ 2.93	1.23	2.52
22	↓ 7.34	20.13	8.88	↑ 29.32	8.44	20.13
23	↓ 2.12	6.46	2.65	↑ 9.77	2.58	6.46
24	↓ 5.76	14.57	7.01	↑ 20.10	6.91	14.57
25	↓ 1.26	3.99	1.55	↑ 4.22	1.93	3.99
26	↓ 0.48	2.42	0.62	↑ 5.21	0.74	2.42
27	0.13	↑ 0.26	0.16	↓ 0.04	0.20	↑ 0.26
28	↓ 8.44	20.38	10.03	↑ 27.53	8.50	20.38
29	↓ 7.69	24.05	8.52	↑ 31.94	9.99	24.05
30	2.32	4.95	↓ 2.14	↑ 7.97	2.90	4.95
31	↓ 3.60	9.53	4.45	↑ 14.10	4.92	9.53
32	1.48	2.51	1.67	↑ 4.93	↓ 1.36	2.51
33	↓ 3.76	11.13	5.86	↑ 18.12	4.69	11.13
34	↓ 1.62	4.38	1.80	↑ 5.52	1.83	4.38
35	↓ 3.35	8.65	3.65	↑ 15.53	4.33	8.65
36	↓ 7.18	20.15	7.84	↑ 29.46	8.97	20.15
37	↓ 5.45	15.53	6.42	↑ 25.83	6.85	15.53
38	1.68	5.00	2.26	↑ 7.05	↓ 1.64	5.00
39	↓ 1.99	4.26	2.77	↑ 8.67	2.80	4.26
40	↓ 6.27	20.40	7.51	↑ 24.96	7.28	20.40
41	↓ 2.23	5.92	2.72	↑ 11.06	3.18	5.92
42	↓ 0.34	1.18	0.36	↑ 1.79	0.82	1.18
43	↓ 5.85	17.98	6.85	↑ 30.57	8.08	17.98
44	↓ 1.15	2.13	1.39	↑ 4.25	1.22	2.13
45	↓ 4.83	15.43	6.48	↑ 24.17	6.64	15.43
46	↓ 1.25	4.46	1.41	↑ 5.51	1.55	4.46
47	↓ 6.94	21.70	8.35	↑ 32.63	9.01	21.70
48	1.93	4.36	↓ 1.65	↑ 8.87	2.40	4.36
49	↓ 0.73	4.96	1.14	↑ 5.35	1.84	4.96

**Table 4:** Head-to-head comparison of SHS and each heuristic. The results indicate the percentage of points per test grid where SHS dominated each particular heuristic.

Grid	SOL	DEG	DOM	MXC	RHO	DOMDEG
Test grid I	84.72%	68.60%	67.48%	68.64%	71.00%	68.12%
Test grid II	84.08%	68.92%	67.64%	68.76%	70.08%	68.08%
Test grid III	83.40%	68.68%	66.80%	69.32%	69.80%	67.64%

**Table 5:** Head-to-head comparison of DHS and each heuristic. The results indicate the percentage of points per test grid where DHS dominated each particular heuristic.

Grid	SOL	DEG	DOM	MXC	RHO	DOMDEG
Test grid I	66.48%	64.84%	59.32%	71.48%	61.15%	60.8%
Test grid II	65.80%	64.84%	59.64%	70.64%	60.56%	60.8%
Test grid III	66.40%	63.84%	58.32%	71.08%	60.92%	59.72%

**Table 6:** Head-to-head comparison of SHS and each heuristic. The results indicate the percentage of saved consistency checks per test grid by using SHS with respect to each particular heuristic.

Grid	SOL	DEG	DOM	MXC	RHO	DOMDEG
Test grid I	1.89%	70.34%	42.36%	60.29%	14.23%	56.87%
Test grid II	1.79%	64.80%	35.90%	59.38%	13.56%	51.47%
Test grid III	1.76%	67.83%	41.93%	60.39%	14.20%	56.62%

**Table 7:** Head-to-head comparison of DHS and each heuristic. The results indicate the percentage of saved consistency checks per test grid by using DHS with respect to each particular heuristic (negative numbers indicate increases in the percentage of consistency checks).

Grid	SOL	DEG	DOM	MXC	RHO	DOMDEG
Test grid I	−9.43%	66.9%	35.74%	55.73%	4.38%	51.9%
Test grid II	−9.51%	60.75%	28.51%	54.70%	3.60%	45.87%
Test grid III	−8.55%	64.45%	35.83%	56.24%	5.2%	52.07%
